# Design, Fabrication and Characterization of Molybdenum Field Emitter Arrays (Mo-FEAs)

**DOI:** 10.3390/mi8050162

**Published:** 2017-05-18

**Authors:** Ningli Zhu, Jing Chen

**Affiliations:** Institute of Microelectronics, Peking University, Beijing 100871, China; ninglizhu@pku.edu.cn

**Keywords:** self-aligned-gate, bulk molybdenum, field emitter array

## Abstract

We report on the fabrication of highly uniform field emitter arrays (FEAs) with an integrated self-aligned extraction gate from bulk molybdenum. All critical dimensions of the emitter tip were determined by a single process step of Inductively Coupled Plasma (ICP) etching. In addition, the height difference between the emitter tip and the gate plane was controlled by the thickness of the SiO_2_ dielectric layer. A 10 µm gate aperture molybdenum-FEAs (Mo-FEAs) at a typical 20 µm pitch with 6 µm height was achieved with 8.4 mA/cm^2^ current density at gate voltages of 110 V and the turn-on field of 1.4 V/µm. These self-aligned Mo-FEAs could be expanded to active larger areas to increase the emission current.

## 1. Introduction

Research on field emitter arrays (FEAs) has been actively pursued with the aim of realizing high current and high current density cathodes, especially for the applications in vacuum electronics [1], display technology [2], and medical X-ray imaging [3]. However, field emission sources are yet to be adopted in demanding applications because of emitter tip radius variation across an array, which results in spatial and temporal variations of emission current [4]. To address the issues of reliability and uniformity, various approaches have been developed over the past few decades to outperform competing cathode designs. Various materials have been investigated as candidates including graphene [5]; carbon nanotubes [6,7,8]; metals such as molybdenum (Mo) [9,10], tungsten [11] and cobalt [12]; metal oxides such as barium oxide (BaO) [13], MoO_x_ [14], and copper (II) oxide [15], etc. Among these materials, Mo has great potential owing to its desirable midgap work function, exceptional thermal and chemical stability, and excellent mechanical properties. Efforts have been made on the fabrication of Mo micro/nanostructures in order to optimize their field emission performance, such as conventional Spindt array with Mo microsized cones [16] and Mo nanowires [17,18]. Pursuits in field emitter array fabrication include sharp emitter tips, alignment, size uniformity, which result in high emitter density, low turn-on field, high field enhancement factor and good emission stability.

For the fabrication process development of the field emitter devices, the standard process of Spindt cone-growth provides the aspect ratio of the emitter cones, which is typically unity and the emission current for stable operation in excess of 12,000 h is 20 µA per tip [19]. For high-emission-current condition, a maximum emission current of 1 mA has been measured from a single Spindt emitter tip [20] for an applied inter-electrode (diode mode) field of 300 V/cm. However, Spindt arrays are subjected to unreliable behavior, which is associated with flashover along the oxide walls and field stress of the tip or the vacuum arcing [21], triggered principally by emission from the triple junction at the emitter base. Post-supported conical tips have been demonstrated to minimize flashover; and, though not immediately associated with overheating or emitter sublimation, enhancement of the emitter’s thermal stability stands to immediately improve the high field operation, particularly when coupled to the use of high temperature stable refractory metals. For design optimization of multiple emitter tips, based on a Line Charge Model, it has been further remarked that Joule and Nottingham heating may well lead to an increase in the emitter temperature [22]. Such a thermal loading is likely the main cause for tip degradation and subsequent decline in emission current with time. Since these interrelated effects are current dependent, the height, location and tip radius of the emitter arrays are controlled to optimize all FEAs for high current and time-stable emission.

In this paper, we report on the fabrication of highly uniform FEAs with an integrated self-aligned extraction gate from bulk Mo. All critical dimensions of the emitter tip were determined by a single process step of Inductively Coupled Plasma (ICP) etching and a new fabrication process of large-area self-aligned gated FEAs has been developed to improve current emission and reliability. Thick gate insulators have been realized to prevent gate dielectric breakdown. In addition, the fabrication of Mo-FEAs has proven to be relatively cost-effective. It is not only to simulate and optimize the field emission tips design, but also to demonstrate the device of Mo-FEAs in experiment that have potential applications such as display and medical X-ray imaging, etc.

## 2. Design and Theoretical Analysis

**Figure 1:**
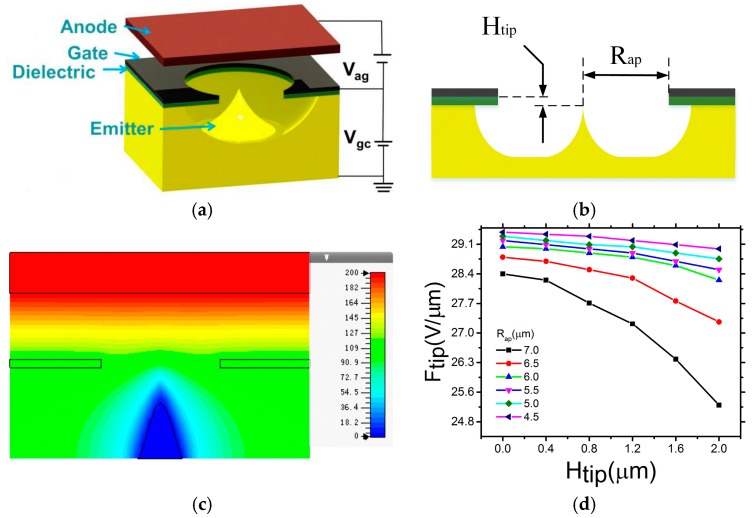
(**a**) Schematic and (**b**) Cross-sectional view of the self-aligned gated field emitter; (**c**) Simulation of the electric field distribution; (**d**) Simulation of the relationship between electric field at the emitter tip and height difference for different gate aperture radii.

Figure 1a depicts the self-aligned gated field emitter. The bulk Mo emitter and the surrounded aluminum (Al) gate are isolated by a dielectric silicon dioxide (SiO_2_) stack. The electric field surrounding the tip is generated by applying a bias voltage between the extraction gate and the emitter. A sharp emitter tip and the presence of the gate in close proximity are necessary to achieve field emission at low voltage.

According to the widely adopted field emission theory for bulk metallic emitters, the emission current can be calculated by the tunneling probability across the potential barrier and the supply rate of electrons to the surface [23]. Based on the Fowler–Nordheim (F–N) equation [24], the emission current density *J* (A/cm^2^) can be expressed as

(1) J=AFtip2∅t(f)2exp[-B∅32Ftipv(f)]

where $F_{\mathrm{tip}}$ is the electric field at the tip apex, ∅ is the emitter surface work function, and $A=1.54\;\text{µ}A\cdot \mathrm{eV}\cdot V^{2}$; $B=6{.83\;\mathrm{eV}}^{-3/2}\cdot V\cdot {\mathrm{nm}}^{-1}$. f is the slope correction factor. t(f) and v(f) are the purely mathematical, slowly varying dimensionless Nordheim elliptical functions. Simply, v(f) is most often set to unity with minimal loss in accuracy and t(f) is expressed as

(2) t(f)=3.79 × 10-5Ftip12∅

In order to optimize the field emission performance, various geometrically critical design parameters must be considered, including the height difference between the emitter tip and the gate plane (*H_tip_*), gate aperture radius (*R_ap_*) and the tip radius (*R_tip_*). Based on the simulation of the effect of radius to electric field, the electric field is increased when tip radius is decreased. Here, we have modeled an *R_tip_* of 35 nm and emitter cone angle of 45°, using CST (Computer Simulation Technology). Space charge effects and resistance limiting have been neglected [25]. The electric field distribution, operated in triode mode (*V_anode_* = 200 V, *V_cathode_* = 0 V, *V_gate_* = 110 V) for a typical emitter is shown in Figure 1c. We find that the electric field at the tip is higher than the bottom. Figure 1d shows how the tip field varies as a function of *R_ap_*, at a gate voltage of 60 V with a range of *H_tip_* from 0 μm to 2 μm. The electric field at the tip ($F_{tip}$) increases with a smaller aperture (*R_ap_*) and smaller height difference (*H_tip_*).

**Figure 2:**
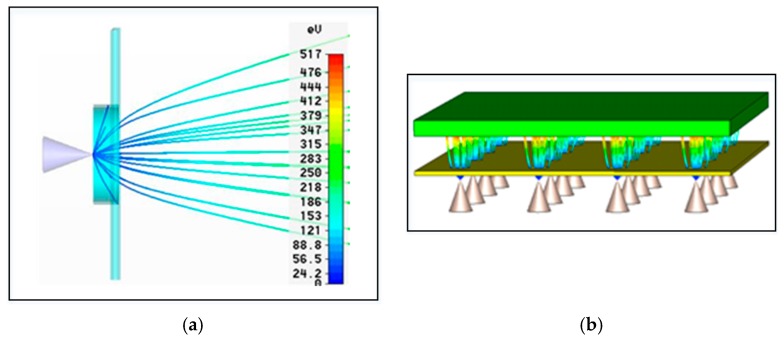
(**a**) Simulation verification of the gate leakage phenomenon and (**b**) Electron trajectories.

Here, we also investigate the average transmission efficiency through a 3D self-consistent ray tracing simulation of the particle trajectories. This can predict the emission current leakage considering unavoidable space charge effects. As shown in Figure 2a, the field emitted current was intercepted by the gate structure, resulting in a gate leakage current of around 20% and the average transmission efficiency of around 80%, which is independently corroborated by our empirical studies. However, due to the lack of an integrated extractor gate, these devices operate at high extraction voltages and 60% of the total emitted current is intercepted by the extraction gate. Furthermore, we will add an integrated gate to improve the robustness of the device, and then higher-current density beam and lower power consumption can be expected.

## 3. Fabrication Processes

**Figure 3:**
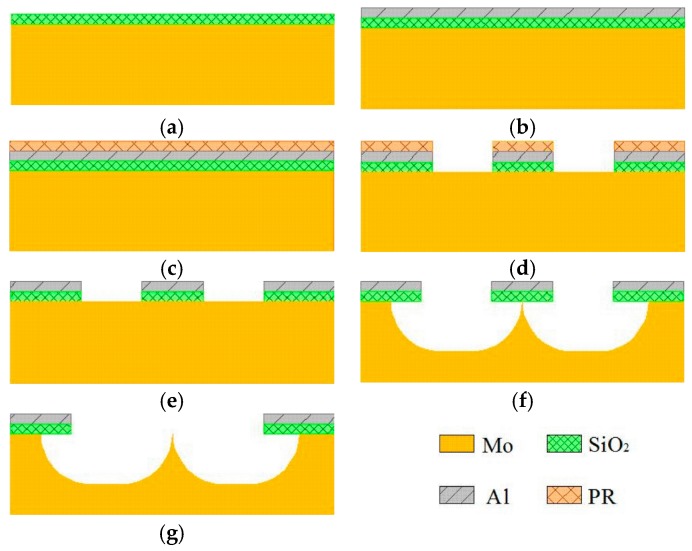
Fabrication process flow of the bulk Mo-FEAs (field emission arrays). (**a**) PECVD SiO_2_; (**b**) Deposition of Al layer; (**c**) Spin coating with PR; (**d**) Lithography and gate-stack etching; (**e**) Strip photoresist; (**f**) ICP etching of Mo emitter cone; (**g**) Ultra-sonication.

The fabrication process flow of self-aligned-gate Mo-FEAs is shown in Figure 3. The whole process requires one single lithography mask. The fabrication steps comprise deposition of SiO_2_ and Al layer, anisotropic etching of gate aperture, isotropic etching of emitter cone and removal of sacrificial SiO_2_/Al stack. The substrate is a 4-inch high purity double-sided polished Mo wafer, with a thickness of 400 μm.

**Figure 4:**
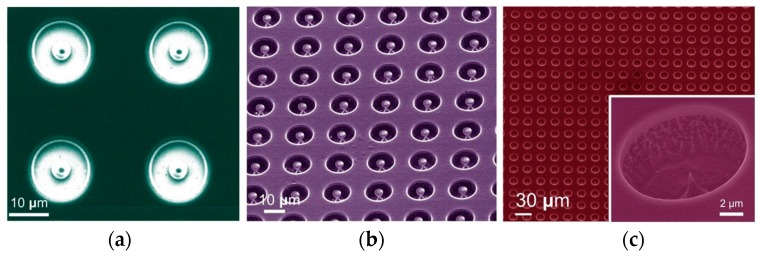
SEM graphs of the device at the various steps of fabrication, including (**a**) Gate-stack etching; (**b**) Inductively Coupled Plasma (ICP) isotropic etching; (**c**) Emitter cone releasing by ultra-sonication. The inset is a finalized single emitter with the self-aligned extraction gate.

Firstly, a SiO_2_ layer with a thickness of 1 μm was deposited as the gate dielectric using plasma enhanced chemical vapor deposition as shown in Figure 3a. Next, a 500 nm Al film was deposited by magnetron sputtering on top of the oxide layer as shown in Figure 3b. This Al film was patterned by dry-etching in CH_3_F plasma as defined by a photoresist mask. The patterned Al film concurrently functions as the SiO_2_ etching hard mask and also latterly as the gate electrode. The patterned SiO_2_/Al/photoresist stacks on the bulk Mo are shown in Figure 3c,d. Figure 4a shows an electron micrograph of the patterned device. The perimeter shadowing of the gate aperture is attributed to the preferential directional bombardment of reactive ions in the dry etching process. The center of the emitter tip, relative to the gate electrode, is thus self-aligned during this process. SEM graphs (false color) of the device at different steps of the fabrication are shown in Figure 4.

The FEA after photoresist stripping is shown in Figure 3e. The surface was flat with no noticeable residues. The Mo FEA tips were etched by SF_6_/Ar dry etching as shown in Figure 3f and Figure 4b. Optimized ICP etcher process conditions, such as pressure, plasma power and gas flows, are reported elsewhere [26]. In the last process step as shown in Figure 3g and Figure 4c, ultrasonic agitation of 15 W was used to remove the sacrificial SiO_2_/Al stack. Here, we define the device yield as the sacrificial stack goes off and the exposed tip is sharp. A device yield of 90% is obtained. The final ultra-sonication principally limits the attainable yield here and future wet-etching is perhaps one good alternative, provided that the selectivity between the gate metal and bulk Mo is high. A completed FEA is shown in Figure 4c, where an enlarged view of a single emitter is shown in the inset. Mo-FEAs with various geometries of patterns were investigated to control the geometry of the resultant tips. For all patterns, the effective radius of the mask geometries was in the range of 2.5–5 μm; gate apertures were in the range of 5–15 μm due to the limitation of fabrication and the cost; and the pitch was in the range of 10–30 μm.

## 4. Experimental Results and Discussions

**Figure 5:**
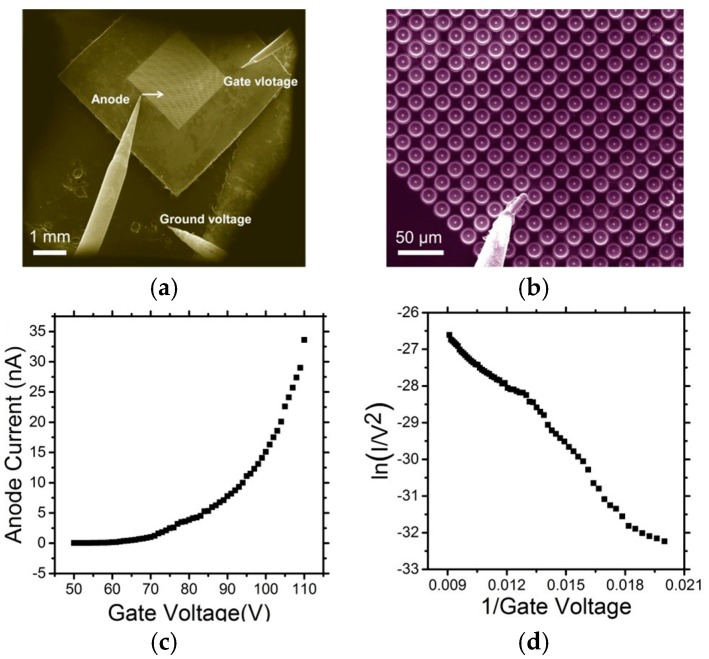
(**a**) Electron micrographs of the measurement configuration consisting of the anode, gate and ground landed electrodes, and (**b**) Magnified view. (**c**) Measured I–V characteristics of one gated Mo field emitter, and (**d**) the corresponding Fowler–Nordheim (F–N) plots.

The emission characteristics as a function of gate voltage were studied in situ using an SEM equipped with four tungsten micro-anode probes, operated with an anode–cathode separation of approximately 50 µm and at a base pressure of 3 × 10^−5^ mbar. The emission current was measured for arrays consisting of approximately 2500 tips. Figure 5a shows the three tungsten probes utilized as the anode, gate and ground probes, respectively. The electron emission from the cathode was investigated using a tungsten probe anode carefully placed adjacent to one Mo tip as shown in Figure 5b. Mo-FEAs of field emitters 6 µm in height with 20 µm pitch were tested. Figure 5c shows typical measured I–V data, and depicts the corresponding F–N plot in Figure 5d. The emission current gives a mean per tip emission of 33.6 nA/tip measured at the gate voltage of 110 V and there is 6.7 nA leakage current at the gate. The average area of each emitter is 20 µm × 20 µm, such that the current density is about 8.4 mA/cm^2^. The F–N plot is to determine whether the device is field emission or not, which uses ln(*I*/*V^2^*) as *y*-axis and 1/*V* as *x*-axis. It is fitted by linear regimes. The average transmission efficiency is approximately 80%, which is consistent with the simulation results as discussed in Section 2.

## 5. Conclusions

For the first time, a new device of self-aligned gated Mo-FEAs that consists of 2500 individual arrays is designed, fabricated and experimentally demonstrated. The field emission density and the turn-on field of the Mo-FEAs with 10 µm gate aperture were measured as 8.4 mA/cm^2^ and 1.4 V/µm, respectively. The bulk Mo-FEAs fabrication processes were developed by using the ICP deep etching technology, which is compatible with the CMOS platform for potential integration with electron sources and control circuits. It is not only to improve the performance of the device, but also to achieve a small size, low-cost and simple device. It has a wide range of potential applications in display and medical X-ray imaging, etc.
